# Split tendon transfers for the correction of spastic varus foot deformity: a case series study

**DOI:** 10.1186/1757-1146-3-28

**Published:** 2010-12-14

**Authors:** Maria Vlachou, Dimitris Dimitriadis

**Affiliations:** 1 Mitera General, Maternity and Children's Hospital, Department of Paediatric Orthopaedics, 6 Erytrou Stavrou & Kifisias street, Marousi 15123, Athens-Greece; 2 Pendeli Children's Hospital, Department of Paediatric Orthopaedics, 8 Ippocratous street, N.Pendeli 15236, Athens-Greece

## Abstract

**Background:**

Overactivity of anterior and/or posterior tibial tendon may be a causative factor of spastic varus foot deformity. The prevalence of their dysfunction has been reported with not well defined results. Although gait analysis and dynamic electromyography provide useful information for the assessment of the patients, they are not available in every hospital. The purpose of the current study is to identify the causative muscle producing the deformity and apply the most suitable technique for its correction.

**Methods:**

We retrospectively evaluated 48 consecutive ambulant patients (52 feet) with spastic paralysis due to cerebral palsy. The average age at the time of the operation was 12,4 yrs (9-18) and the mean follow-up 7,8 yrs (4-14). Eigtheen feet presented equinus hind foot deformity due to gastrocnemius and soleus shortening. According to the deformity, the feet were divided in two groups (Group I with forefoot and midfoot inversion and Group II with hindfoot varus). The deformities were flexible in all cases in both groups. Split anterior tibial tendon transfer (SPLATT) was performed in Group I (11 feet), while split posterior tibial tendon transfer (SPOTT) was performed in Group II (38 feet). In 3 feet both procedures were performed. Achilles tendon sliding lengthening (Hoke procedure) was done in 18 feet either preoperatively or concomitantly with the index procedure.

**Results:**

The results in Group I, were rated according to Hoffer's clinical criteria as excellent in 8 feet and satisfactory in 3, while in Group II according to Kling's clinical criteria were rated as excellent in 20 feet, good in 14 and poor in 4. The feet with poor results presented residual varus deformity due to intraoperative technical errors.

**Conclusion:**

Overactivity of the anterior tibial tendon produces inversion most prominent in the forefoot and midfoot and similarly overactivity of the posterior tibial tendon produces hindfoot varus. The deformity can be clinically unidentifiable in some cases when Achilles shortening co-exists producing foot equinus. By identifying the muscle causing the deformity and performing the appropriate technique, very satisfying results were achieved in the majority of our cases. In three feet both muscles contributed to a combined deformity and simultaneous SPLATT and SPOTT were considered necessary. For complex foot deformities where the component of cavus co-exists, supplementary procedures are required along with the index operation to obtain the best result.

## Introduction

Varus foot is often secondary to cerebral palsy and split tibialis anterior (SPLATT) or posterior tibialis tendon transfers (SPOTT) are commonly performed to correct the deformity. In both procedures the distal part of the tendon is splitting longitudinally, half of the tendon is detached from its medial insertion and is reattached to the lateral side of the foot [1]. The goal of the semi-transfers is to affect the muscle-tendon complex in a way that it neither inverts nor everts the foot maintaining thus its stability and flexibility.

**Figure 1:**
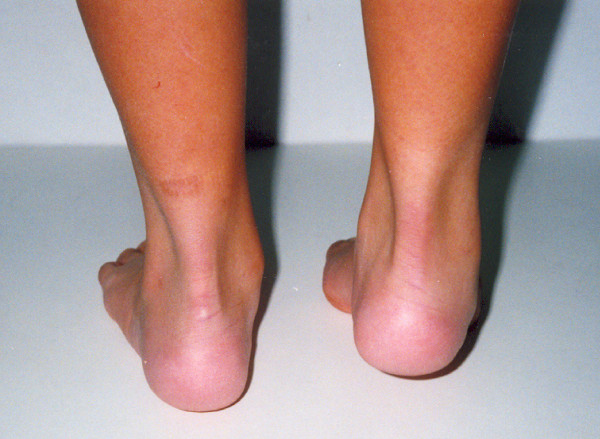
**A 12 year-old female patient with Rt equinovarus hind foot deformity on weight bearing position**.

The SPLATT as described by Hoffer et al [2] corrects supination and varus deformity of the midfoot secondary to spasticity of the anterior tibial muscle. Equinovarus hindfoot deformity is most common in children with spastic hemiplegia and is caused by spasticity of the posterior tibial muscle that very often is associated with weakness of the peroneal muscles and tightness of the heel cord [Figure 1].

The reported clinical outcomes of SPLATT and SPOTT have been generally good but have been considerably varied [2-9]. SPLATT was first described by Kaufer et al [10] and was popularized by Green et al [4] and Kling et al [6] as a technique that balances the hind part of the foot and maintains the plantar flexion power, but it should be applied only to patients from 4 to 6 years of age due to the potential risk of converting the foot to a valgus deformity in children younger than 4 years. Prerequisites of split tendon transfers is the ability or the potential ability for walking. Contraindications include a fixed bony deformity, and severe contraction mainly concerning the anterior tibialis, as the transferred semi-tendon can not reach the cuboid bone.

## Materials and methods

Written parental permission was obtained to allow the use of information held in the hospital records to be used in this review as Institutional Review Board (IRB) does not exist in our country.

The cohort of the study consisted of 48 consecutive ambulant or potentially ambulant patients (52 feet) with spastic paralysis and dynamic equinovarus foot deformity that underwent split anterior (SPLATT) or split posterior (SPOTT) tendon transfer.

The hemiplegic patients were 32, the diplegic 12 and the quadriplegic 4.

**Figure 2:**
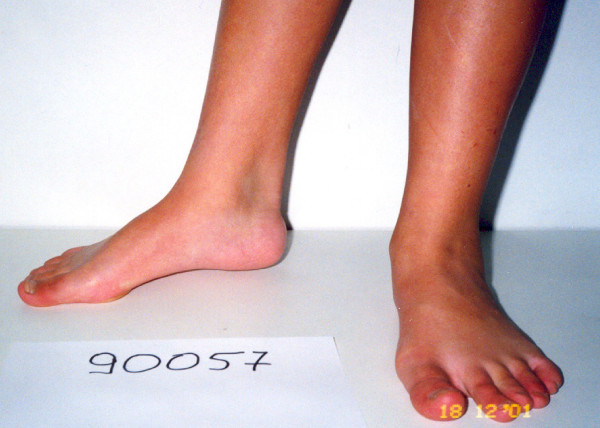
**Lateral view of the same foot with mild cavus component**.

**Figure 3:**
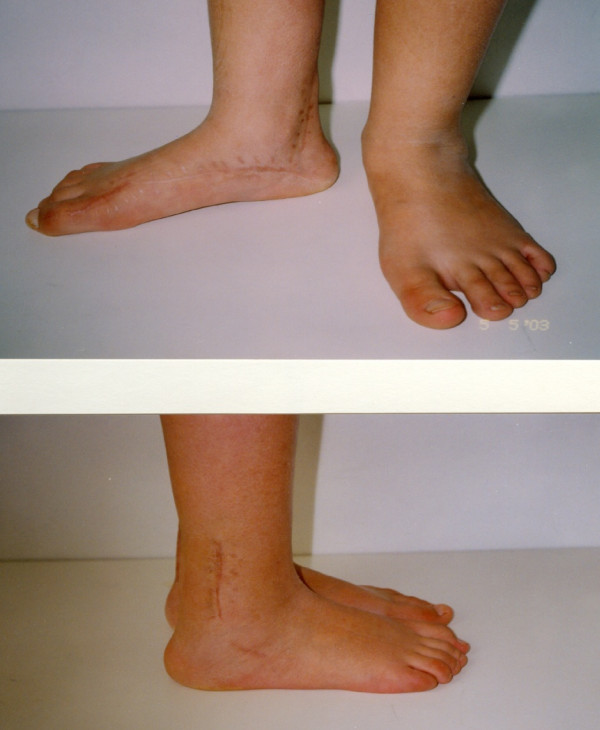
**Postperative lateral views of the patient after plantar soft tissue release, Achilles lengthening and concomitant split posterior tendon transfer**.

Our inclusion criteria were: 1. ambulatory or potentially ambulatory patients with cerebral palsy, 2. age no less than 6 years at the time of the operation, 3. varus deformity of the hind foot during gait (stance and swing phase), 4. flexible varus foot deformity, and 5. follow-up at least 4 years. Eigtheen feet presented equinus hind foot deformity due to triceps shortening. According to the deformity, the feet were divided in two groups (Group I with predominant forefoot and midfoot inversion and Group II with predominant hindfoot varus). The deformities were flexible in all cases in both groups. The first group consisted of 11 patients (11 feet, 9 female-2 male) all unilateral, 10 of them presenting hemiplegia and one quadriplegia. They also presented prominent forefoot and midfoot inversion due to overactivity of the anterior tibial tendon (AT), associated with a mild cavus component [Figure 2, 3]. Patients in this group underwent the SPLATT (Hoffer's procedure). The second group consisted of 34 patients (38 feet, 24 female-10 male). The hemiplegic patients were 20, the diplegic 11 and the quadriplegic 3. They presented prominent varus hindfoot which persisted during the entire gait cycle due to the overactive PT. Patients in this group underwent the split PT tendon transfer (Green's procedure). Eighteen feet presented also equinus hind foot deformity, requiring concomitant Achilles cord lengthening. Three patients (3 feet), two hemiplegic and one diplegic that were not included in the groups, underwent both procedures because both muscles contributed to a combined deformity and simultaneous SPLATT and SPOTT were performed. Clinical evaluation was based on the inspection of the patients while standing and walking, the range of motion of the foot and ankle, callus formation and the foot appearance using the clinical criteria of Hoffer et al [1] in Group I and of Kling et al [6] in Group II. According to Hoffer [1], the result was considered very good when there was no deformity postoperatively, total foot contact on the ground and proper shoe wearing. Satisfactory was considered when there was mild varus, valgus or equinus deformity, small foot contact and overnight braces were used. Poor was considered when there was overcorrection, undercorrection or equinus > 5° and braces were available. According to Kling [6] excellent results were graded when the child managed to walk with a plantigrade foot, without fixed or postural deformity, in a regular shoe having no callosities. Patients and parents were pleased with the result and no brace was required post-operatively. Results were graded good in children who walk with less than 5° varus, valgus, or equinus posture of the hind foot, wearing regular shoes, having no callosities and were satisfied with the outcome. Feet with recurrent equinovarus deformity, or overcorrected into a valgus or calcaneovalgus deformity were considered as poor results.

The position of the hind foot was evaluated according to the criteria of Chang et al [11] for the surgical outcome. Severe varus was defined when the hind foot was in > 10° varus and additional operations were required, mild varus when the hind foot was in 5° to 10° of varus and no additional operation was required, neutral when the hind foot was in neutral position or in less than 5° of varus or valgus, mild valgus when the hind foot is in 5° to 10° of valgus with no additional operations and severe valgus when the hind foot was in more than 10° of valgus and additional operations were required.

### Surgical procedures

In SPLATT, the first incision exposed the insertion of the tendon which was split longitudinally as far as through the musculotendinous junction. The medial half of the tendon was left attached to the first metatarsal and first cuneiform, but the lateral half was detached from its insertion. The split lateral half of the tendon was passed subcutaneously into the incision made over the cuboid and then was inserted into the holes made in the bone and either sutured to itself under moderate tension or if the length of the stump was not sufficient, anchoring was carried out with any other technique (pull-out wire, anchoring to the periosteum, etc.). For SPOTT, four separate incisions were used according to Green et al [4]. The first incision two centimetres long was positioned over the insertion of the posterior tibialis tendon on the navicular. The distal end of the tendon was identified and its sheath was opened.

The tendon was split longitudinally and the plantar half was dissected from its insertion. The free end was grasped and the tendon was split longitudinally as far proximally as possible. The second incision begun at the level of the medial malleolus and continued for approximately six centimetres. The free half of the tendon was transferred into the proximal incision and the longitudinal split in the tendon was continued to the musculotendinous junction. A third incision is made directly posterior to the lateral malleolus beginning at the proximal tip of the malleolus and continuing proximally. The peroneus brevis was identified and its sheath was split longitudinally. The distal stump of the split posterior tibial tendon in the second incision was threaded into a tendon-passer that passed the split portion directly posterior to the tibia and fibula and anterior to all the neurovascular and tendinous structures so as to enter laterally to the opened sheath of peroneus brevis. The fourth incision was made along the peroneus brevis and begun distal to the lateral malleolus and continued distally just proximal to the insertion of the peroneus brevis on the base of the fifth metatarsal. The distal part of the sheath was opened and the split posterior tibial tendon was sutured to the peroneal brevis tendon onto the cuboid by fish-mouth technique. The tension should be adjusted so that the hind part of the foot will rest in neutral position, by holding the foot in neutral and pulling hard on the posterior tibial tendon and slightly reducing the pull. The heel-cord lengthening was usually performed prior to the procedure and a long cast was applied with the knee extended and the foot in neutral position. The patient could bear weight on the cast as tolerated and four weeks later the cast was changed and a short walking cast was applied. If the patient was able to dorsiflex the foot and ankle to neutral, no postoperative brace was used.

## Results

**Figure 4:**
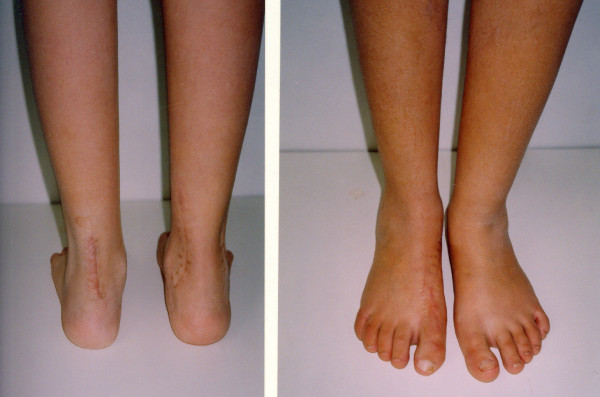
**Postoperative anterior and posterior views of the same patient in six years follow-up**.

Evaluation of the results was carried out using the clinical criteria of Hoffer [1] in group one and Kling and Kaufer [6] in group two (Tables 1, 2). In the former, very good results were obtained in 8 feet and satisfactory in 3. In the later one, 22 feet were excellent, 12 good and 4 poor. The 3 feet that underwent simultaneously both of the procedures presented 1 excellent and 2 satisfactory results. In the first group due to mild cavus foot component supplementary operations were performed at the same time with the index procedure [Figure 3, 4]. Plantar soft tissue releases (open release of the plantar aponeurosis+release of the plantar muscles from their insertion into the calcaneus) were performed in 11 feet, transcutaneous flexor tenotomies in 8, and Jones procedure in 5 feet (Table 2).

**Table 1:** Results. Number in parentheses is the total number of the feet and the percentage of the results according to the involvement.

Group II	Excellent (20)	Good (14)	Poor (4)
***Hemiplegia (22)***	20 (90,9%)	2 (9,09%)	-

***Diplegia (12)***	-	12 (100%)	-

***Quadriplegia (4)***	-	1 (25%)	3 (75%)

**Group I**	Excellent (8)	Satisfactory (3)	Poor

***Hemiplegia (10)***	8 (80%)	2 (20%)	-

***Diplegia***	-	-	-

***Quadriplegia (1)***	-	1(100%)	-

**Both SPLATT-SPOTT**	Excellent (2)	Satisfactory or Good (1)	Poor

***Hemiplegia (2)***	2 (100%)	-	-

***Diplegia (1)***	-	1 (100%)	-

***Quadriplegia***	-	-	-

**Table 2:** Supplementary operations performed concomitant with the index operation (SPLATT).

Supplementary operations	Feet (No)
	Group I
**Plantar soft tissue releases**	11

**Transcutaneous flexor tenotomies**	8

**Jones (transfer of the long toe extensor tendon to the neck of the 1^st ^metatarsal)**	5

The mean range of motion at the last follow-up was 10-20° of dorsiflexion, 30-40° of plantarflexion, 25-30° of foot inversion and 15-20° of foot eversion. No overcorrection or undercorrection was reported.

In the second group, 23 feet presenting concomitant cavus foot component that underwent supplementary operations performed at the same time with the index operation. Plantar soft tissue releases were performed in 15 feet, Jones procedure in 5, long extensor tendons transfer to the metatarsals in 2, as well as transcutaneous flexor tenotomies in 23 feet (Table 3). It has also been required concomitant Achilles cord lengthening in 18 feet due to the equinus position of the hind foot. None of the feet presented mild or severe valgus postoperatively, while 4 feet presented severe varus deformity and underwent calcaneocuboid fusion sixteen and eighteen months after the index operation. The mean value of mild varus was (-14,5 ± 12,2°) and concerning the feet with the hind foot in neutral position the mean value was 5.0 ± 7.4°.

**Table 3:** Supplementary operations performed concomitant and after the index operation (SPOTT).

*Supplementary operations*	Feet (No)
	Group II
**Transcutaneous flexor tenotomies**	23

**Achilles cord lengthenings**	18

**Plantar soft tissue releases**	15

**Jones**	5

**Extensor tendons transfer to the metatarsals**	2

***After index operation***	

**Calcaneocuboid fusion (Evans)**	4

The results in patients with hemiplegic pattern were better and significantly different than the diplegic and quadriplegic ones (p = 0.005), by using the chi-square analyses as statistical significant involvement at p < 0.01 in the second group (Table 1). All patients with an excellent result were brace free at the last follow-up with significant improvement in gait, able to walk with plantigrade feet, use of regular shoes and parent's satisfaction with the outcome. The patients with good results continued to use a night brace (AFO). All of them had good correction of the hind foot equinus and the ankle was able to dorsiflex to at least 90°. The patients presenting a poor result required continued bracing because of the severe varus residual deformity, appearing excessive weight bear on the lateral border of the foot and having painful callosities. These patients required further foot realignment (calcaneocuboid fusion).

## Discussion

**Figure 5:**
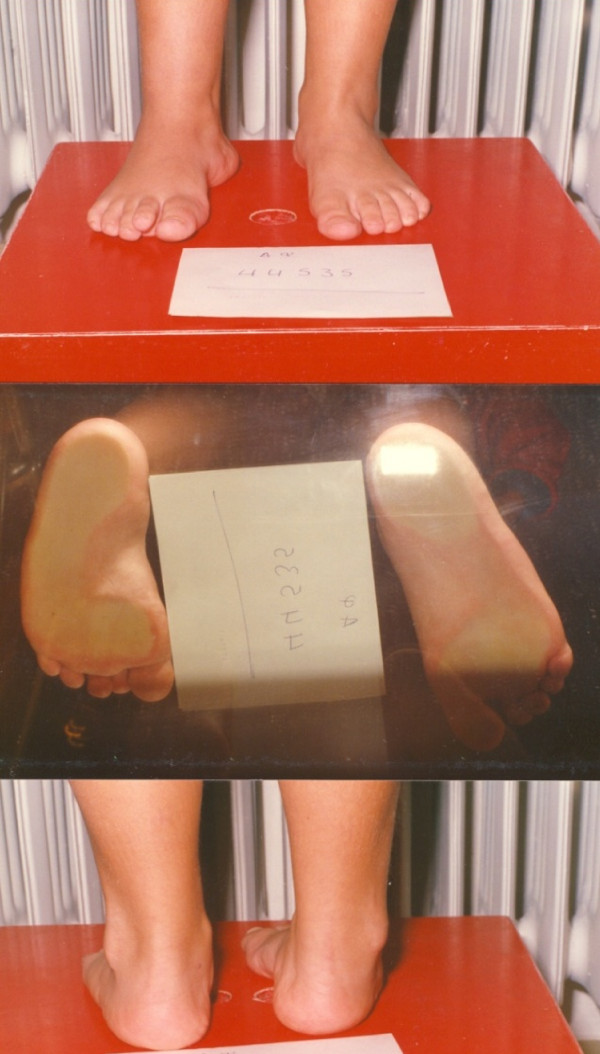
**A 10 year-old male patient with Rt varus hind foot and mild cavus deformity**.

**Figure 6:**
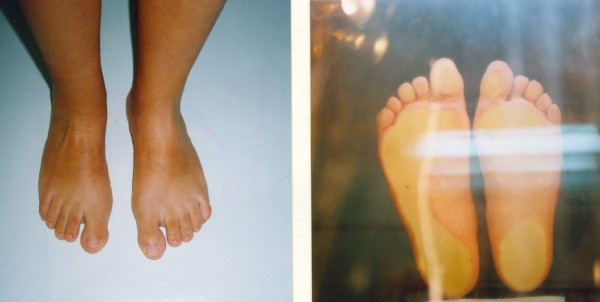
**Increased pressure areas are demonstrated on podoscope**.

**Figure 7:**
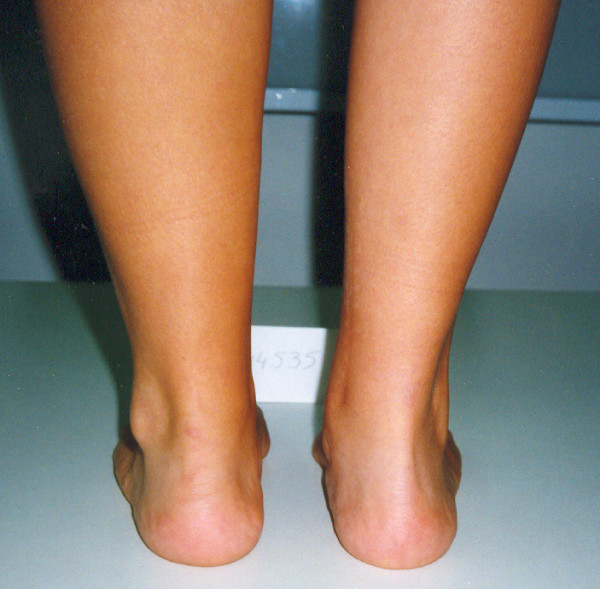
**Postoperative neutral position of the hind foot after plantar soft tissue release and split posterior tendon transfer**.

**Figure 8:**
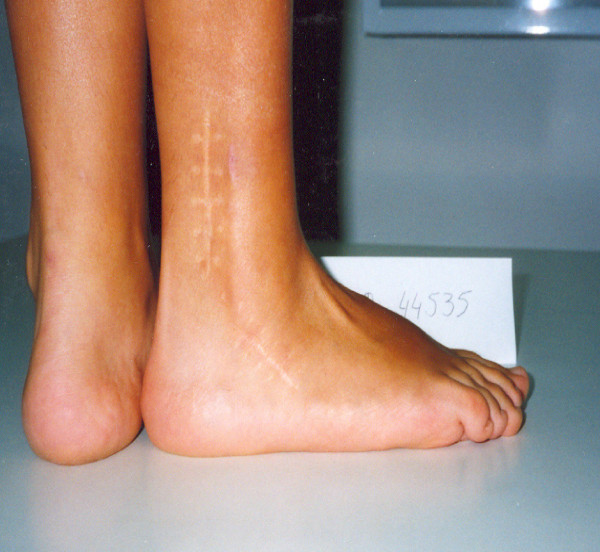
**Final result in a five year follow-up**.

**Figure 9:**
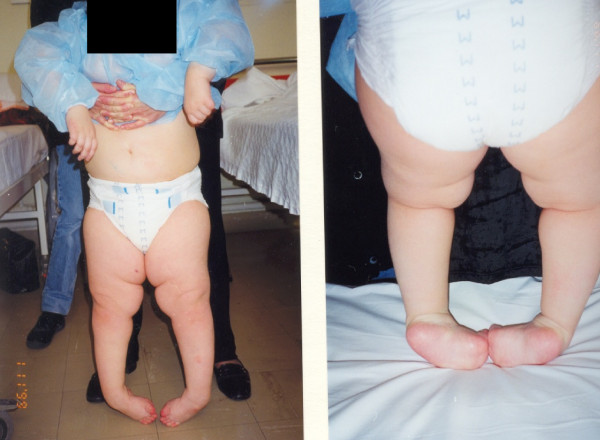
**A seven-year old female patient with severe equinovarus hind foot deformity with inability to stand and walk**.

**Figure 10:**
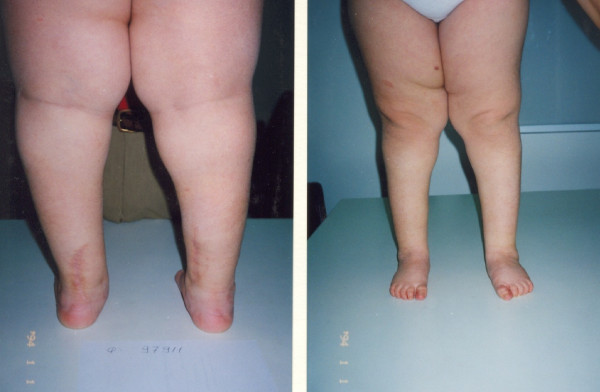
**Postoperative posterior and anterior views after Achilles lengthening and concomitant bilateral split posterior tendon transfer**.

**Figure 11:**
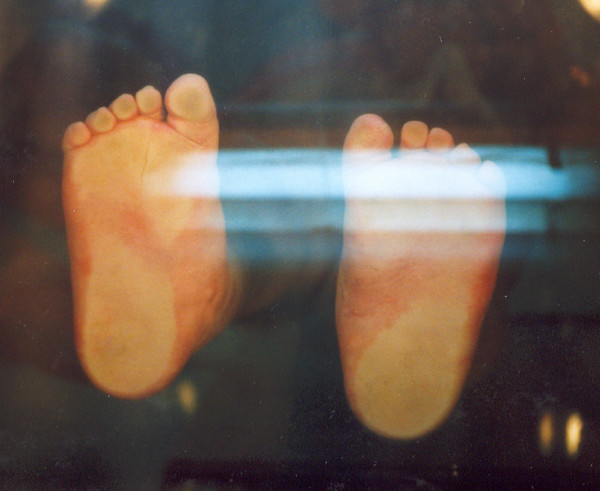
**Final result on podoscope**.

**Figure 12:**
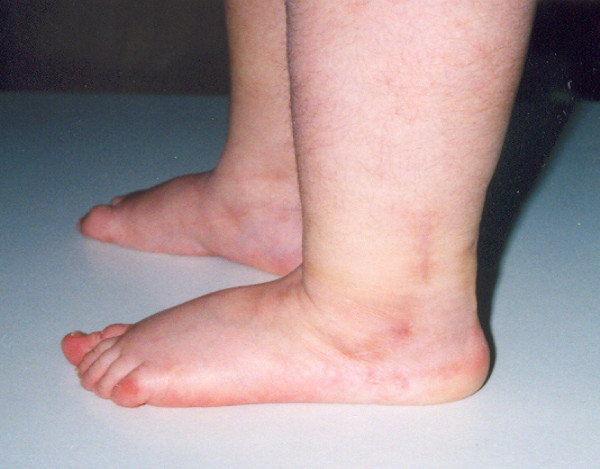
**Lateral view in a 4 year follow-up**.

It is generally accepted that overactivity of the AT is responsible for varus-inversion forefoot deformity, whereas the overactivity of PT causes equinovarus hind-foot deformity. Between these two conditions the second is much more common. However, the cause of the deformity can not be clinically identified in some cases, especially when Achilles shortening co-exists. The use of dynamic electromyography and gait analysis can be helpful, but it can not be available in every Institution. Twenty-seven out of thirty-eight feet in the second group presented concomitant cavus foot component and underwent supplementary operations performed concomitant with the index operation. Plantar soft tissue release was the most common out of these procedures, as the release constitutes a keystone procedure for lengthening the shortened base of the foot, and its contribution to the successful outcome for the correction of the cavus component cannot be overemphasized [Figure 5, 6]. Our results in hemiplegic patients were better and significantly different than the diplegic and quadriplegic ones, indicating that the underlying neurologic impairement affect the results of the surgery [Figure 7, 8]. One of our prerequisite for split tibialis tendon transfers was the ability of the patients for walking or the potential of standing and ambulation [Figure 9, 10, 11 and 12]. The simple lengthening of the posterior tibialis tendon weakens the muscle, and if the tendon and the heel cord are lengthened, then plantar-flexion strength is significantly reduced.

All reports regarding split tendon transfers showed favourable results [4-8,11,12] with our study supporting the same. Two factors that should be considered to perform the procedures are: the flexibility of the deformity and the dorsiflexion of the ankle to at least 5°- 10° beyond neutral. The fixed bony deformity prevents complete correction of the equinovarus position of the foot and if it co-exists, a bone procedure should be considered before the tendon transfer, to prevent persistent varus. In the present series, only flexible deformities passively corrected were included. The extensor tendons transfer to the metatarsals aimed to improve the metatarsophalangeal dysfunction, to enhance ankle dorsiflexion and in association with the transcutaneous flexor tenotomies in several toes to correct the clawing. Although overcorrection is more difficult to treat [13] we did not observe any, while the poor results in SPLATT included severe varus deformity in 4 feet that required calcaneocuboid fusion. One of our inclusion criteria was the age of the patients at the time of the surgery to be more than 6 years, as it is an important factor for the final outcome. Ruda and Frost [14] reported that after intramuscular posterior tendon lengthening in 29 patients, the reccurence in varus observed in two, being less than 6 years of age. Lee and Bleck [15] reported a reccurence of 29% in patients less than 8 years at the time of the operation, as the spastic muscle tends to retain its contractile properties even if it is weakened or transferred at an age younger than 8 years. In conclusion, the rapid bone growth in children that underwent split tibialis tendon transfers in less than 6 years of age, may lead to reccurence. We selected a follow-up period of more than 4 years as the failure rate increases with the postoperative period and the final results can be estimated only after the skeletal growth [11].

Residual varus deformity in our series were attributed to technical intraoperative errors in balancing the tension between the medial and lateral tendon halves. Four feet underwent bone procedure for the correction of the hindfoot deformity at a later stage. In 3 feet, some technical difficulty was encountered in suturing the split posterior tibial tendon to the peroneus brevis, as the split half being short. Although we performed concomitant intramuscular lengthening of this part of the tendon so as to be sufficient for transfer, we do not recommend it as the tendon loose more of its power.

**Figure 13:**
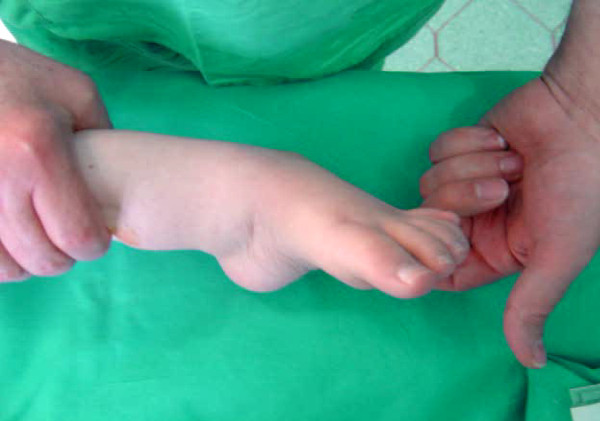
**Intraoperative view of a six year old hemiplegic patient with varus forefoot deformity**.

**Figure 14:**
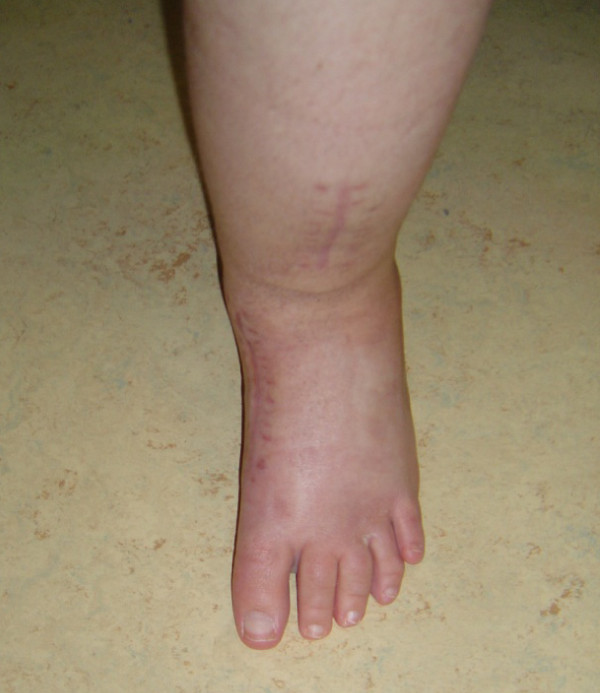
**Anterior view of the Rt foot after split anterior tibial tendon transfer in a 4 year follow-up**.

Additionally, in 3 feet both muscles contributed to a combined deformity, which was defined only intraoperatively, and therefore simultaneous SPLATT and SPOTT were satisfactorily performed [Figure 13, 14].

The purpose of doing split tendon transfers as opposed to whole tendon transfers in children with cerebral palsy has been considered to be more effective, as it distributes equally the muscle power, eliminating the possibility of residual deformity or overcorrection. Anterior transposition or rerouting of the posterior tibial tendon has been previously described [16] but calcaneus deformity may be a result in spastic muscles, by converting the PT into an ankle dorsiflexor [17].

The anterior transfer of the posterior tibial muscle through the interosseous membrane is an attractive procedure as it removes the actual deforming force and balances the weak or absent anterior tibial and peroneal muscles, but is effective in patients with nonspastic paralytic equinovarus deformities of the foot [18] A continuously spastic posterior tibial muscle will maintain its spasticity when transferred and if a heel cord lengthening is performed concomitant with the anterior transfer of the posterior tibialis, a calcaneus deformity may likely result.

In the majority of our cases, the deforming force was successfully determined preoperatively and the final results justified the applied operative procedure. In complex deformities, other supplementary procedures may be required to achieve the best possible outcome.

## Competing interests

The authors declare that they have no competing interests.

## Consent

Written informed consent was obtained from the patient for publication of this case report and accompanying images. A copy of the written consent is available for review by the Editor-in-Chief of this journal.

## Authors' contributions

MV: Analysis and interpretation of data, preparation of the manuscript,

acquisition of pictures and additional materials, corresponding author.

DD: Approved the final manuscript.

All authors read and approved the final manuscript.
